# Comprehensive evaluation of biostimulants on leaf-level physiology, water use efficiency, and tuber quality in potato (*Solanum tuberosum* L.) under drought stress

**DOI:** 10.3389/fpls.2026.1837304

**Published:** 2026-07-06

**Authors:** Muhammad Ahsan Asghar, Karen la Cour Jørgensen, Thayná Mendanha dos Santos, Hanne Lakkenborg Kristensen, Merete Edelenbos, Mesfin Tsegaye Gebremikael

**Affiliations:** Department of Food Science, Aarhus University, Agro Food Park, Aarhus, Denmark

**Keywords:** biomass, biostimulants, drought, morphology, photosynthesis, tuber quality

## Abstract

The pressing need for sustainable agriculture and food security has led to increased interest in biostimulants (BS) to mitigate abiotic stresses such as drought. This study provides a comprehensive evaluation of five commercial BS (Vesta, Humifirst, Acadian, SilicaPower, and Crop-Set) on leaf-level physiology, water use efficiency (WUE), tuber yield, and tuber quality in potato under drought stress. Drought stress was applied at the onset of tuber initiation (51 days after planting) and lasted for 7 days followed by a 4-day recovery period. Drought stress significantly decreased leaf relative water content, photosynthetic parameters, and flavonol index compared to non-drought. The yield and glucose content were 26% and 21% lower, and the sucrose content was 59% higher in the tubers of the drought-stressed plants than in non-drought-stressed. BS effects were generally limited and dependent on water conditions, with significant interactions observed for WUE parameters. Under drought conditions, Vesta and SilicaPower increased intrinsic WUE by 36% and 30%, respectively, while instantaneous WUE increased by 25% with both Vesta and Humifirst and by 33% with SilicaPower. Under non-drought conditions, Acadian led to a 42% increase in intrinsic WUE and a 60% increase in instantaneous WUE. However, these effects were not consistently accompanied by improvements in photosynthesis, biomass, or yield. Overall, this comprehensive assessment indicates that tested BS exert limited effects on potato plant biomass and tubers yield under drought stress, with their primary influence restricted to increased leaf-level WUE and changes in tuber composition rather than providing broad drought stress alleviation at whole plant level.

## Graphical Abstract

**Figure:**
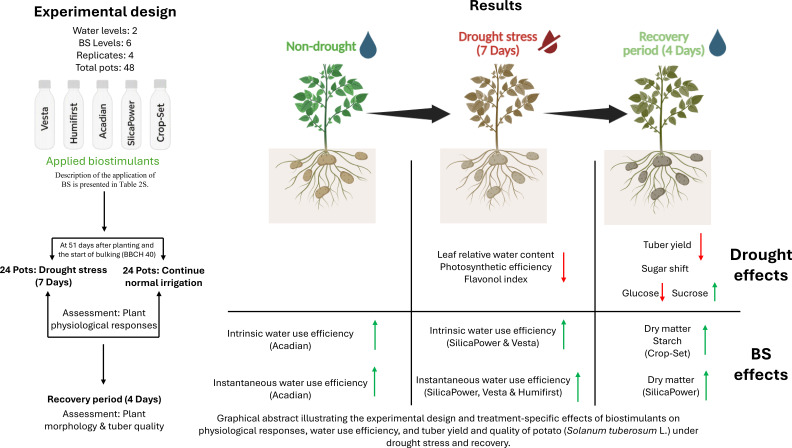
The abstract should ideally be structured according to the IMRaD format (Introduction, Methods, Results and Discussion). Provide a structured abstract if possible. If your article has been copyedited by us, please provide the updated abstract based on this version.

## 1 Introduction

In a changing climate, the challenge of keeping up with the growing demand for food is a crisis that cannot be ignored. Among climatic extremes, drought stress is one of the most serious challenges, drastically affecting crop growth and yield, and ultimately impacting sustainable agriculture (Ghadirnezhad Shiade et al., 2023; More et al., 2023). It results in crop dehydration, which may eventually lead to reduced plant growth and development (Merwad et al., 2018; Mansour et al., 2021). Water deficiency adversely impacts mineral nutrition (nutrient intake, transportation, and uptake) and metabolism, resulting in reduced leaf area and altered partitioning and assimilation into organs (Semida and Rady, 2014). Drought stress disrupts plant growth and metabolism by reducing water status and turgor. It decreases stomatal conductance and photosynthesis while increasing reactive oxygen species (ROS), which can cause oxidative damage. Water deficit suppresses CO_2_ assimilation and carbon fixation, alters carbohydrate metabolism, and shifts dry matter partitioning (Bashir et al., 2021). Drought can also impair photosystem function and photochemical efficiency, lowering chlorophyll a fluorescence (Gervais et al., 2021). Plants have therefore evolved mechanisms that can absorb and take up more water and activate antioxidative defense systems to minimize the devastating effects of drought stress on cells, including reducing leaf area and stomatal conductance, developing deeper roots, and producing more osmolytes than needed (Obidiegwu, 2015).

Potato plays a crucial role in global food security, ranking as the fourth most widely cultivated crop for human consumption. As a major staple food, it is rich in vitamins, calories, and nutrients (Obidiegwu, 2015). However, it is predicted that future droughts will reduce global potato yields by 18–32% (Hijmans, 2003). Like other plants, potatoes activate key physiological and molecular mechanisms of drought tolerance, including improved water use efficiency (WUE), osmotic adjustments, and carbon partitioning into tubers (Aliche et al., 2020). However, these protective mechanisms may not be sufficient under severe or prolonged drought. Alternative strategies are, therefore, necessary to enhance plant stress resilience and ensure food security in a changing climate.

Biostimulants (BS) are increasingly promoted as sustainable and eco-friendly biosolutions to overcome biotic and abiotic stresses, including drought, and to enhance crop resilience (Wadas and Dziugieł, 2020; Ma et al., 2022; Alowaiesh et al., 2024). By modulating plant metabolism and signaling, BS have been shown to induce osmotic adjustment, stimulate antioxidant defense and root growth, improve WUE, and possibly modify carbon partitioning to storage organs, such as the potato tuber, during drought (Han et al., 2021; Di Sario et al., 2025). Several BS have been tested for improving drought stress tolerance in potatoes. Results show that ascorbic acid, benzoic acid, and salicylic acid enhance plant resilience by improving plant growth under drought stress when applied to the foliage (Alowaiesh et al., 2024). Fulvic and humic acids extracted from leonardite, seaweed extracts of *Ascophyllum nodosum* and *Ecklonia maxima* have also been shown to enhance the yield of marketable tubers by improving photosynthesis and resistance against abiotic stress (Wadas and Dziugieł, 2020). Moreover, a liquid suspension BS (MX42SEK^®^) appears to positively affect chlorophyll content, leaf area, and leaf assimilation, ultimately boosting potato tuber weight by an average of 33% across cultivars (Salvage et al., 2024). In another study, dry matter, vitamin C, and starch concentration, as well as the tuber yield, increased following inoculation of seed potatoes with bacteria (Groundfix^®^) and humic substances (Agriful^®^) (Andrejiová et al., 2023). Spraying melatonin onto potato foliage during drought improved leaf relative water content, free amino acid content, sugar content, antioxidant activity, and cell membrane stability. In contrast, the contents of carotenoids, proline, methylglyoxal, hydrogen peroxide, malondialdehyde and abscisic acid decreased (El-Yazied et al., 2022). Taken altogether, most studies focus on evaluating the efficacy of BS on physiological traits, yield and tuber quality separately. However, less is known about the extent to which BS can mitigate drought stress and associated yield reductions in potato, particularly under field soil conditions. While previous studies have examined physiological, yield, or quality responses separately, fewer studies have evaluated these traits simultaneously within a single experimental framework. Therefore, this study provides a comprehensive evaluation of BS effects across leaf-level physiology, WUE, tuber yield, and tuber quality in potato under drought stress.

This study aimed to explore the effects of commercially available BS, such as microorganisms, substances derived from organic matter decomposition, seaweed extracts, inorganic compounds, and plant extracts (**Table 1**), on mitigating drought stress in potatoes. The effects of drought stress were studied during tuber initiation and bulking, growth phases characterized by strong sink demand (Rai and Dong, 2025), and plant responses were assessed using a comprehensive approach encompassing leaf physiology and tuber quality. The hypotheses were twofold: 1) BS modulate stomatal behavior and leaf gas exchange to increase WUE, enabling potato plants to assimilate more carbon per unit water lost rather than merely increasing absolute photosynthetic rates, under both drought and non-drought conditions, and 2) BS can regulate seed composition under drought stress by promoting osmolyte (dry matter, starch, soluble sugars) accumulation, thereby contributing to osmotic adjustment and buffering tuber development and quality and ultimately the overall plant biomass.

**Table 1:** Origin of biostimulants and claimed effects.

Biostimulant	Vesta	Humifirst	Acadian	SilicaPower	Crop-Set
Type	Microbial biostimulant	Humic–fulvic acid biostimulant	Seaweed extract biostimulant	Silicon biostimulant containing orthosilicic acid	Fermentation-based biostimulant with micronutrients
Key composition stated by the producer	Suspension of >5,400 living microorganisms extracted from fermented seaweed, microbial metabolites, and humic extract	Extract of humic (12 %) and fulvic acids (3 %) made from American Leonardit	Seaweed (Ascophyllum nodosum) extracted with potassium hydroxide. The extract contains biactive acids produced by algae, polysaccharides including fucose, mannitol, monosaccharides, amino acids, organic acids, betaines, and macro- and micronutrients (Zn, Mg, Fe)	Liquid containing pure colloidal micro-SiO_2_ with 3% orthosilicic acid	A mixture of yucca palm extract (Yucca schidigera), a bacterial fermented extract without alive bacteria, and addition of sulfur sulfate (1.2% S), copper sulfate (0.2% Cu), iron sulfate (0.6% Fe), and manganese (1.5% Mn)
Claimed main effects	Restores soil microbiology and increases root growth. Increases nutrient cycling efficiency and improves nutrient use efficiency. Increase plant vigor and uniformity	Improves soil aggregation and ICEC, and germination and root growth. Improves nutrient uptake (P, Fe, Mn, Zn, Cu). Increases root and plant growth, enhancing yields and crop quality	Increased root growth and improved plant establishment. Improved plant vigor and plant growth enhancing yield and crop quality	Strengthens cell walls, improves nutrient uptake and distribution (Ca, Mn), promotes growth of root hairs, and increases antioxidative defence compounds	Improves nutrient uptake and partitioning, and stimulates plant metabolic processes (root growth, vegetative growth, reproductive growth) through greater availability of essential nutrients. Improves crop vigor, yield and marketable yield, including crop uniformity
Claimed stress tolerance effects	Increases tolerance to heat and water stress. Improves photosynthesis	Increases resistance to drought, salinity, and cold stress as a result of increased enzymatic and non-enzymatic defence, increased compatible solute production, and changed ion balance	Enhances tolerance to drought and cold stress, linked to upregulation of defence-related genes and pathways in the plant	Protects the plant from abiotic stress such as drought, salinity, heat and cold stress	Activates the plant defence mechanisms, increases photosynthetic pigments and gas exchange under drought stress
Reference	https://sovitae.com/products/	https://www.nature.com/articles/s41598-020-63925-5#Sec9	https://acadianplanthealth.com/resources/articles/raising-the-bar-on-crop-quality	SilicaPower: https://www.plantosys.com/nl/producten/silicapower	https://www.bj-agro.dk/biostimulanter/cropset-en-biostimulant-til-at-oege-udbyttet-i-kartofler
			https://acadianplanthealth.com/the-solution/asm		

## 2 Materials and methods

### 2.1 Soil collection, growing conditions and application of BS

This study was conducted from March 23 to May 24, 2022, at the greenhouse of the Department of Food Science at Aarhus University (AU), Denmark. The soil was collected from the 0-0.25 m topsoil layer of a field at AU Research Station in Auning, Denmark (56°44′ N, 10°34′ E). The soil texture consisted of 6.5% clay, 3.2% silt, 87% sand, and 3.4% organic matter, with a soil pH of 5.9. The experiment was conducted in 3.5 L pots containing a mixture of 10% sphagnum peat and 90% sandy soil, supplemented with fertilizers. The fertilizer application rate was set at 100 kg N/ha, 30 kg P/ha, and 200 kg K/ha based on the recommended practice of conventional potato production for the variety in Denmark. One seed potato (*Solanum tuberosum* L.) was planted per pot of the early table variety ‘Arielle’ (European cultivated potato database, 2024). Tubers had an average weight of 57.4 g and a diameter of 4.9 cm at the widest point perpendicular to the tuber’s length. To minimize variability associated with the use of seed tubers, all tubers were carefully selected to ensure uniformity in size, weight, and physiological status (e.g., sprouting condition and absence of defects). The tuber was planted at a depth of 8 cm with germination buds facing upward. The pots were placed in the greenhouse and watered with 100 mL of water three times a week until all plants emerged, and subsequently with 200 mL of water three times a week. A 15 h photo period with LED lights was applied at a light intensity of at least 150 µmol·m^-2^·s^-1^ photosynthetic photon flux density (PPFD). The day and night temperatures were 24 °C and 18 °C, respectively. Drought stress was applied at tuber initiation, 51 days after planting and lasted for 7 days. Five commercial BS, namely Vesta™, Humifirst™, Acadian™, SilicaPower™, and Crop-Set™, were applied at planting as a coating on the tuber (Vesta), in the planting hole before planting (Humifirst, Crop-Set) and/or as a soil and or foliar spray during growth and development. The BS were applied at a rate and frequency recommended by each company that provided the BS (**Supplementary Table 1**).

### 2.2 Experimental design, drought stress and plant recovery4

**Figure 1:**
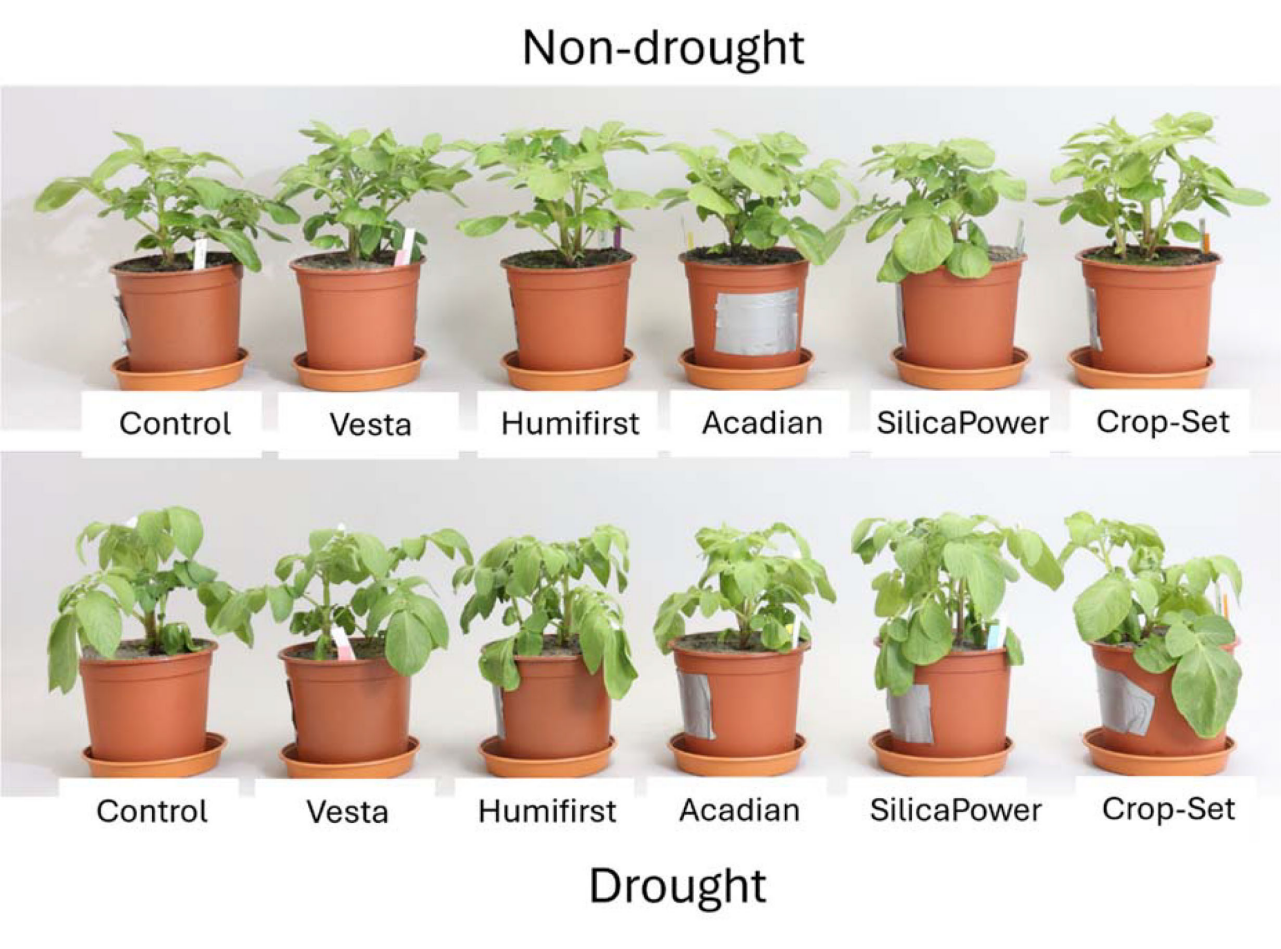
Non-drought (top) and drought-stressed (bottom) potato plants after 7 days of drought stress. The plants were treated with Vesta, Humifirst, Acadian, SilicaPower, and Crop-Set (biostimulants) before the onset of drought stress at tuber initiation (day 51 after planting).

The experiment was set up using a complete randomized block design, with two fixed factors: (i) water status with two levels: non-drought-stress versus drought-stress, and (ii) BS with six levels: control (no BS), Vesta, Humifirst, Acadian, SilicaPower, and Crop-Set. A total of 48 pots were included in the experiment (2 drought stress levels * 6 BS levels * 4 replicates). All pots were irrigated until tuber initiation, 51 days after planting and the start of bulking (BBCH 40). Then, half of the pots were drought-stressed for 7 days where the plants showed wilting symptoms (**Figure 1**). The remaining pots (non-drought-stressed plants) were irrigated as before. Both the non-drought-stressed and the drought-stressed plants were placed on Phenospex Drought Spotters to automatically irrigate the non-drought-stressed plants or abstain from irrigating the drought-stressed plants. The direct effects of drought stress and BS treatments were determined at the end of the stress period (7-day drought stress). The measured parameters included leaf relative water content, leaf temperature, intrinsic WUE, and instantaneous WUE. In addition, gas exchange traits such as net photosynthetic rate, stomatal conductance, transpiration rate, and intercellular CO_2_ concentration were assessed. Chlorophyll fluorescence parameters, including electron transport rate and photosystem yield, were also measured. Furthermore, the leaf chlorophyll index, flavonol index, and nitrogen balance index were determined. The post-drought recovery effects were assessed after 4 days of rewatering by measuring fresh and dry weight of leaves and stems, leaf area per plant, number of stems, and number of initiated tubers. In addition, the number, fresh weight, and dry matter of developed tubers, tuber yield, as well as tuber quality traits, were determined. During the recovery period, plants were watered as before drought stress.

### 2.3 Leaf relative water content

The leaf relative water content was determined on one fully developed leaf per pot using the following formula (Turner, 1981):


Leaf Relative Water Content=(FW−DW)/(TW−DW)×100

FW is the fresh weight of the leaf, TW is the turgid weight after rehydrating the leaf for 24 h, and DW is the dry weight of the leaf after oven-drying at 80 °C for 24 h.

### 2.4 Gas exchange and chlorophyll fluorescence

The net photosynthetic rate, transpiration rate, stomatal conductance, leaf temperature, and chlorophyll fluorescence were determined on one fully developed leaf per pot, following a previously established method (Kňazovický et al., 2026). The leaf was placed in a 4 cm² leaf cuvette, and gas exchange was measured using a portable gas exchange fluorescence system (Walz GFS-3000, Walz GmbH, Effeltrich, Germany) equipped with an integrated red-blue LED light source (Walz 3055-FL). The instrument started at 500 µmol·m^-2^·s^-1^ and slowly increased to 2000 µmol·m^-2^·s^-1^ to avoid photoinhibition. The data was collected at the PPFD level. The flow rate was 750 mL·min-^1^, the CO_2_ concentration was 450 ppm, and the cuvette temperature was 25 °C. The relative humidity in the cuvette was maintained at 65-75% to keep the vapor pressure deficit below 10.0 Pa/kPa. For chlorophyll fluorescence, the plants were moved to a dark room and dark-adapted for 30 min. An amplitude modulation fluorimeter (PAM-2500, Walz Eiffeltrich, Germany), equipped with a fiber-optic probe positioned perpendicular to the leaf surface at a fixed distance, was used to assess the photosystem yield and electron transport rate (µmol electrons m^-^² s^-^¹) on the adaxial surface of the same leaves as for the gas exchange measurements. A PPFD of 3500 µmol m^-^² s^-^¹ was used as a saturating flash with a duration of 1 s.

The intrinsic WUE was calculated as follows:


Intrinsic Water Use Efficiency=Net Photosynthetic Rate/Stomatal Conductance

The instantaneous WUE was calculated as follows:


Instantaneous Water Use Efficiency=Net Photosynthetic Rate/Transpiration Rate

Intrinsic WUE reflects the balance between carbon assimilation and stomatal conductance, representing the efficiency of carbon gain relative to stomatal opening. In contrast, instantaneous WUE relates carbon assimilation to transpiration rate and therefore reflects the efficiency of carbon gain per unit of water lost through transpiration. While intrinsic WUE is primarily driven by stomatal regulation, instantaneous WUE integrates both stomatal and atmospheric influences on water loss.

### 2.5 Leaf chlorophyll index, flavonol index and nitrogen balance index

The leaf chlorophyll and flavonol indices were measured using an optical leaf clip meter (Dualex 4 Scientific, FORCE-A, Orsay, France) (Padilla et al., 2014). The nitrogen balance index was calculated as follows:


Nitrogen Balance Index=Chlorophyll Index/Flavonol Index

### 2.6 Plant morphology

Morphological measurements were taken at the end of the recovery period. The aboveground biomass was harvested by cutting the lateral stems (branches) with leaves at soil surface and removing all aboveground material. The number of lateral stems and leaves was counted, and the FW of each part was recorded. The leaf area per plant was determined by scanning all leaves and analyzing the images using the LI-3100C Portable Leaf Area Meter (LI-COR, Lincoln, NE, USA) by following the previously published protocol (Kňazovický et al., 2026). The DW was determined after oven-drying for 24 h at 80 °C.

### 2.7 Tuber yield and quality

The tuber yield was determined after harvesting the aboveground biomass. The pot was turned upside down, the soil gently removed, and the initiated and developed tubers were counted. If the tuber weight was more than 5 g after being gently cleaned with a slightly moist cloth, it was counted as a developed tuber. From the FW of the developed tubers, tuber yield per plant was determined. Tubers of comparable size (weight, length, and diameter; see **Table 2S**) were selected for constituent analyses, as the dry matter, sugar, and starch contents change with tuber size during bulking (Kolbe and Stephan-beckmann, 1997). A cube without skin was made from each tuber. From this cube, smaller cubes of approximately 5 × 5 × 5 mm were cut, carefully mixed, and frozen in liquid nitrogen, freeze-dried, crushed, and ground into a fine powder in a bead mill (Retsch Milling Mixer MM 200). The dry matter content was determined as (DW/FW) * 100. Soluble sugars (glucose, fructose and sucrose) and starch were extracted using a previously published protocol with some modifications (Viola et al., 2007). For extraction, 50 mg of powder was mixed with 950 µL of 80% ethanol, heated at 80 °C for 1 h with periodic vortexing, centrifuged at 16,000 g for 10 min at 1 °C, and then re-extracted. The two supernatant fractions were combined, reduced to dryness in a SpeedVac at 50 °C, and then re-suspended in 1000 µL of sterile deionized water. The sample was kept at 80 °C for 30 min and vortexed periodically. The samples were centrifuged at 16,000 g for 5 min at 1 °C, filtered through a 0.45 µm filter directly into a 2 mL glass vial, and stored at -20 °C until analysis. For starch extraction, the pellet was dried in a SpeedVac for 20–30 min at 50 °C, resuspended in 1,000 µL of sterile deionized water, and kept for 15 min to allow the pellet to absorb the water before centrifugation at 16,000 g for 5 min at 1 °C. The supernatant was discarded, and the washing was repeated. The washed pellet was then resuspended in 450 µL deionized water, heated to 100 °C for 1 h to gelatinize the starch, and cooled to room temperature. To convert starch to glucose, 50 µL of 1 M sodium acetate at pH 4.5, containing 100 U mL^-1^ of α-amyloglucosidase (diluted in sodium acetate), was added, and the solution was incubated for 18 h at 45 °C. A few drops of Lugol solution (Lugol: deionized water, 1:10) were added after incubation to test whether all starch had been converted to glucose (no blackening of the solution). The solution was centrifuged at 5,000 g for 5 min at 1 °C, then filtered through a 0.45 µm filter directly into a 2 mL glass vial and stored at -20 °C until analysis. Sugars were analyzed by high-performance anion-exchange chromatography with pulsed amperometric detection (HPAEC-PAD) after appropriate dilutions of the samples. The analysis was carried out on a CarboPac PA1 column using isocratic elution with 200 mM NaOH (Dionex, ICS 6000, Sunnyvale, CA) at a flow rate of 0.25 mL min^-1^. The sugars were identified by peak addition and quantified using calibration curves of authentic standards. The concentration of starch is expressed as glucose equivalent.

#### 2.7.1 Total plant dry biomass

Total plant dry biomass was estimated by combining aboveground and belowground dry mass. The DW of stems and leaves was measured directly after oven-drying, as described above. Tuber dry mass was calculated by multiplying the total tuber fresh yield per plant by the corresponding tuber dry matter percentage, determined by freeze-drying. Total plant dry biomass was then calculated as the sum of aboveground dry weight and tuber dry mass on a per-plant basis.

### 2.8 Statistical analysis

A two-way ANOVA was applied to assess the effects of the two main factors (drought and BS) and their interaction. If no significant interaction was observed, the *post hoc* analysis was performed for the main factors. However, if the interaction was significant, the *post hoc* analysis used the least significant difference (LSD) test at the 5% significance level to examine the interaction effects of the two factors. All measurements were taken in four replicates and reported with standard deviations. For the statistical analysis, mean values were applied when repeated measures were taken on the plant. The analysis was conducted using Statistics 8.1 software (Analytical Software, Inc.).

## 3 Results

### 3.1 Phenological appearance of plants

The drought-stressed plants showed severe drought symptoms after 7 days, compared with the non-drought-stressed plants (**Figure 1**). The leaves had lost turgor and were wilted after exposure to drought stress. However, no apparent visual differences were observed between the non-drought-stressed and the drought-stressed plants following BS applications.

### 3.2 Water use efficiency

**Figure 2:**
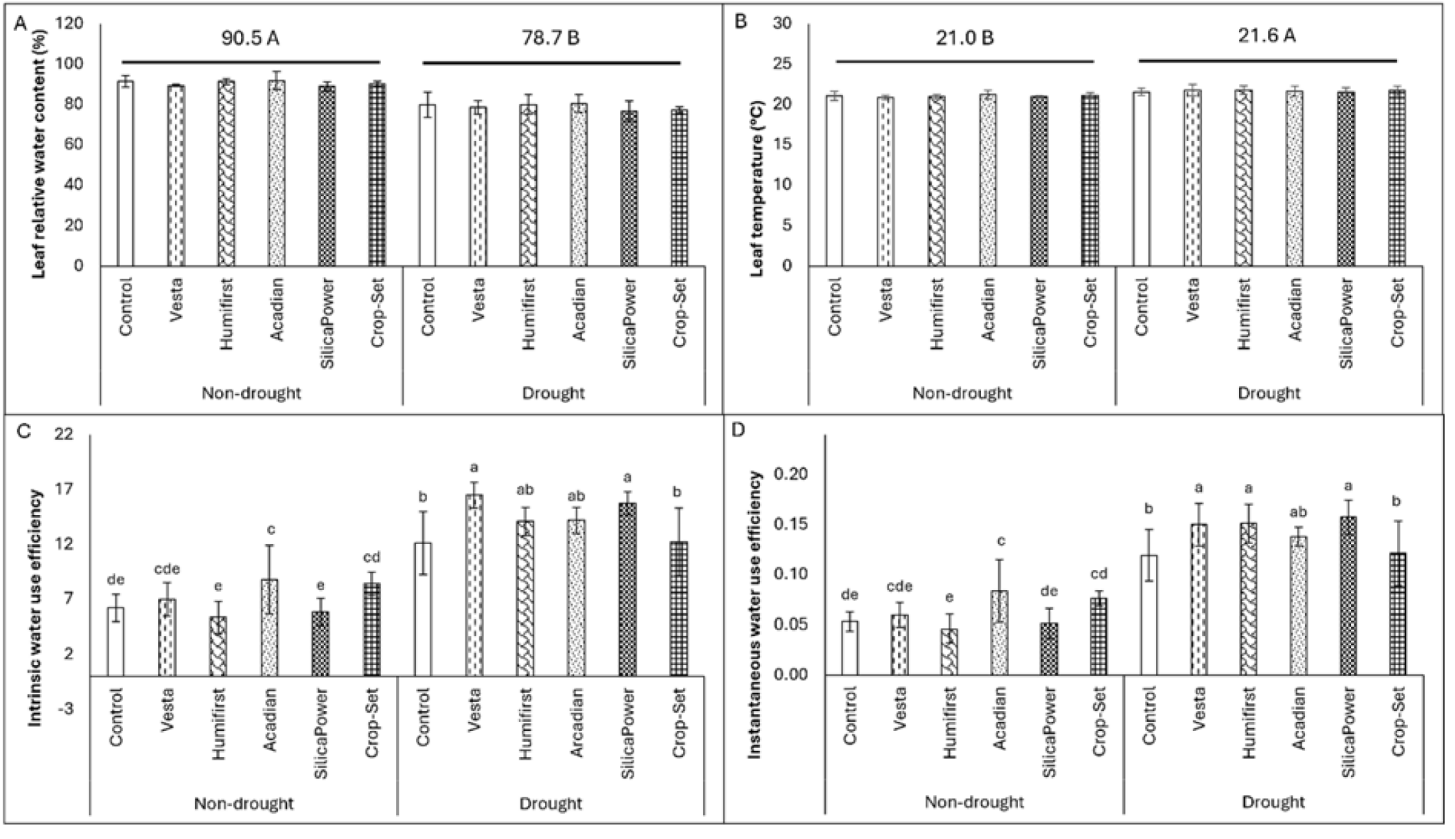
The effect of biostimulants on plant water relations **(A, B)** and water use efficiency **(C, D)** of potato plants after 7 days of drought stress, relative to non-drought-treated plants. The plants were treated with Vesta, Humifirst, Acadian, SilicaPower, and Crop-Set before the onset of drought stress. Data represent the means ± standard deviation (n = 4). A two-way ANOVA was used to evaluate the main effects of drought and biostimulant treatment, as well as their interaction. Horizontal bars with different capital letters indicate significant differences between non-drought and drought stress conditions averaged across biostimulant treatments based on post hoc comparisons (LSD test) at P = 0.05. Different lowercase letters indicate significant differences among treatment combinations (drought × biostimulants) based on post hoc comparisons (LSD test) at P = 0.05.

Leaf relative water content was 13% lower following drought stress (*p* = 0.00) (**Figure 2A**), and there was no effect of significant effect of any of the applied BS on leaf relative water content. However, there was no effect of the BS on leaf relative water content under non-drought and drought-stressed conditions (*p* = 0.3), and there was no interaction of BS and drought stress (*p* = 0.9). The leaf temperature increased under drought stress, from 21 to 21.6 °C. However, BS did not affect leaf temperature, and there was no interaction between drought stress and BS (**Figure 2B**; **Table 3S**).

The intrinsic WUE increased by 96% under drought stress, from 6.9 to 14.1 (**Figure 2C**). Under non-drought-stress conditions, a 42% increase in intrinsic WUE was observed with Acadian application as compared to control (without BS). While under drought stress conditions, application of Vesta and SilicaPower resulted in an increase in the intrinsic WUE by 36 and 30%, respectively, as compared to control (without BS) (**Figure 2C**). Similarly, the instantaneous WUE increased by 60% with application of Acadian under non-drought conditions as compared to control (without BS) and by 25% with Vesta and Humifirst and 33% with SilicaPower under drought-stress conditions as compared to control (without BS) (**Figure 2D**). There was a significant interaction between drought and BS for intrinsic WUE (*p* = 0.005) and instantaneous WUE (*p* = 0.008) (**Table 3S**).

### 3.3 Gas exchange

**Figure 3:**
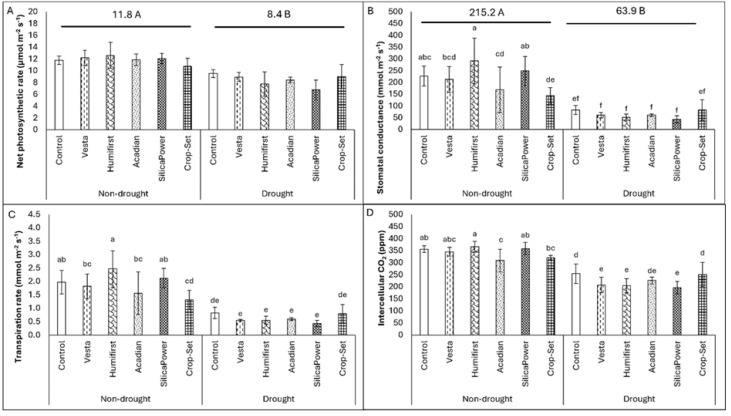
The effect of biostimulants on gas exchange parameters **(A–D)** of potato plants after 7 days of drought stress, relative to non-drought-treated plants. The plants were treated with Vesta, Humifirst, Acadian, SilicaPower, and Crop-Set before the onset of drought stress. Data represent the means ± standard deviation (n = 4). A two-way ANOVA was used to evaluate the main effects of drought and biostimulant treatment, as well as their interaction. Horizontal bars with different capital letters indicate significant differences between non-drought and drought stress conditions averaged across biostimulant treatments based on post hoc comparisons (LSD test) at P = 0.05. Different lowercase letters indicate significant differences among treatment combinations (drought × biostimulants) based on post hoc comparisons (LSD test) at P = 0.05.

The net photosynthetic rate was substantially reduced (29%) by drought stress (**Figure 3A**). In contrast, there was no effect (*p* = 0.5) of BS on net photosynthetic rate, and the interaction between drought and BS was also non-significant (*p* = 0.11) (**Table 3S**). Upon drought stress, significant reductions were observed in stomatal conductance (70%), transpiration rate (66%), and intercellular CO_2_ (35%) as compared to non-drought. As with net photosynthetic rate, the main effect of BS application during growth did not yield substantial changes in the gas exchange parameters (**Figures 3B–D**). However, there were significant interactions (*p* = 0.02, *p* = 0.02, and *p* = 0.01) between drought stress and BS for stomatal conductance, transpiration rate, and intercellular CO_2_, respectively (**Table 3S**).

### 3.4 Chlorophyll fluorescence

**Figure 4:**
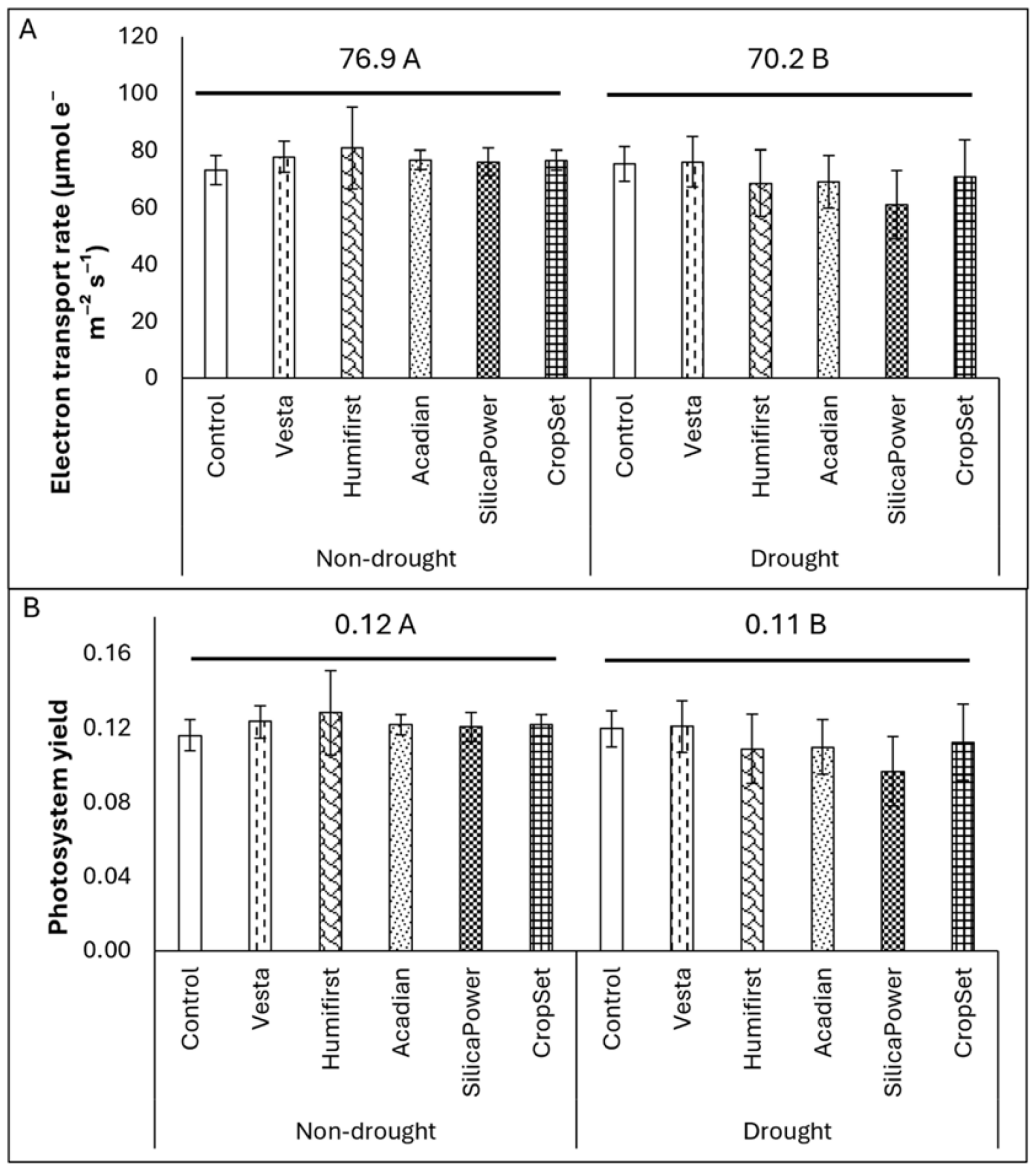
The effect of biostimulants on electron transport rate **(A)** and photosystem yield **(B)** of potato plants after 7 days of drought stress, relative to non-drought-treated plants. The plants were treated with Vesta, Humifirst, Acadian, SilicaPower, and Crop-Set before the onset of drought stress. Data represent the means ± standard deviation (n = 4). A two-way ANOVA was used to evaluate the main effects of drought and biostimulant treatment, as well as their interaction. Horizontal bars with different capital letters indicate significant differences between non-drought and drought stress conditions averaged across biostimulant treatments based on post hoc comparisons (LSD test) at P = 0.05.

A considerable reduction in electron transport rate (9%) and photosystem yield (8%) was observed following drought stress. In contrast, the potato plants did not exhibit significant changes in chlorophyll fluorescence parameters after exposure to BS (**Figures 4A, B**). Both the chlorophyll fluorescence parameters (electron transport rate and photosystem yield) exhibited similar results, with the interaction between drought and BS being non-significant (*p* = 0.4) (**Table 3S**).

### 3.5 Leaf pigments

**Figure 5:**
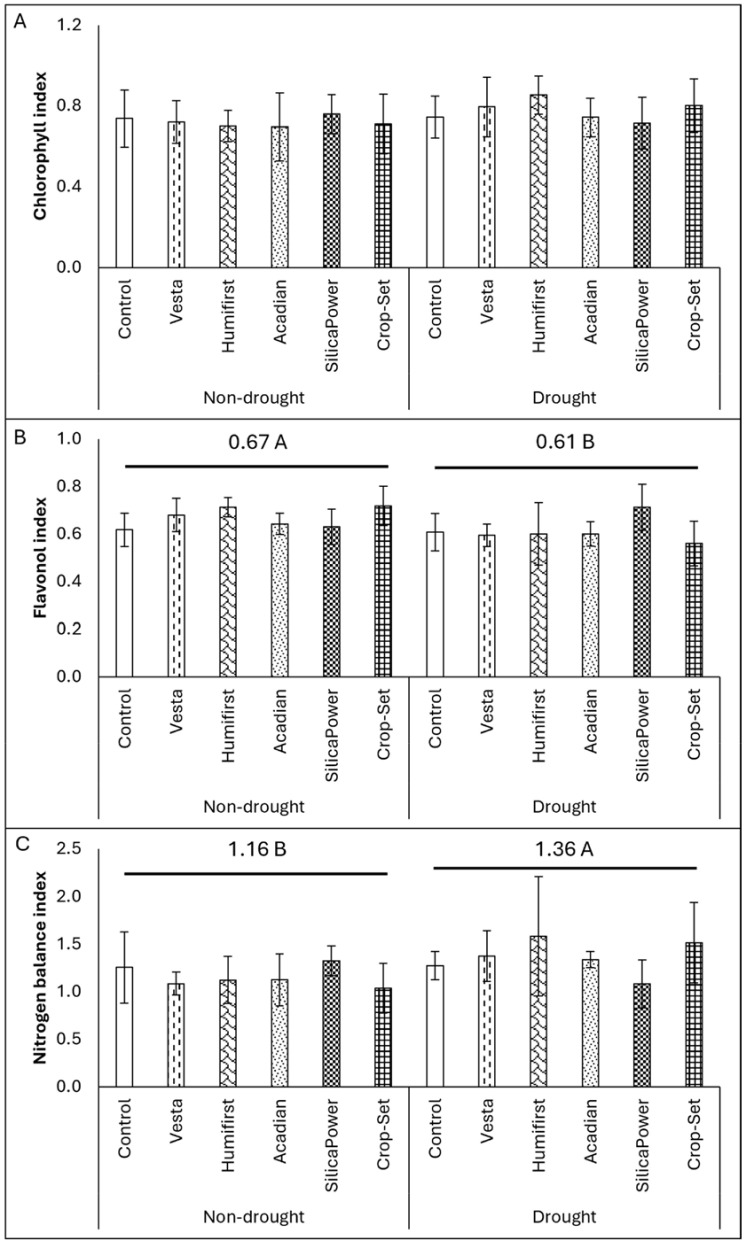
The effect of biostimulants on chlorophyll index **(A)**, flavonol index **(B)**, and nitrogen balance index **(C)** of potato plants after 7 days of drought stress, relative to non-drought-treated plants. The plants were treated with Vesta, Humifirst, Acadian, SilicaPower, and Crop-Set before the onset of drought stress. Data represent the means ± standard deviation (n = 4). A two-way ANOVA was used to evaluate the main effects of drought and biostimulant treatment, as well as their interaction. Horizontal bars with different capital letters indicate significant differences between non-drought and drought stress conditions averaged across biostimulant treatments based on post hoc comparisons (LSD test) at P = 0.05.

Surprisingly, the chlorophyll index was unaffected by drought stress (*p* = 0.1). Similarly, BS did not affect the chlorophyll index (*p* = 0.9), and the interaction between drought and BS was also non-significant (*p* = 0.6) (**Figure 5A**).

Following drought stress, the potato plants had significantly lower (9%) flavonol index (*p* = 0.01), while the index remained unchanged when the plants were treated with BS (*p* = 0.5). Interestingly, the impact of drought stress was still dependent on BS, as evident by the significant interaction (*p* = 0.03) (**Figure 5B**). Contrary to the flavonol index, the nitrogen balance index was substantially enhanced (17%) after drought stress. As with other physiological parameters, no significant change in nitrogen balance index was observed after application of BS (**Figure 5C**). For the nitrogen balance index, the interaction between drought and BS was also non-significant (**Table 3S**).

### 3.6 Plant morphology

There were no effects of drought stress on the fresh weight of leaves and stems, dry weight of leaves and stems, leaf area per plant and number of stems per plant after the recovery period (**Table 2**). Similarly, the addition of BS did not affect the plant morphology of the potato plants. The interaction between drought and BS was significant for the fresh weight of leaves and stems (*p* = 0.053) and the dry weight of leaves and stems (*p* = 0.03) (**Table 2**).

**Table 2:** The effect of biostimulants on the morphology of non-drought-stressed and drought-stressed potato plants.

Drought stress	Biostimulants	Fresh weight of leaves and stems	Dry weight of leaves and stems	Total plant dry biomass	Leaf area cm^2^ plant^-1^	No. of stems
D	BS	g	g	g/plant^-1^		
Non-drought	Control	50.7 ± 2.2	5.3 ± 0.3	20.0 ± 4.2	915 ± 31	2.5 ± 0.2
	Vesta	53.1 ± 2.6	5.5 ± 0.5	16.48 ± 5.0	956 ± 55	2.5 ± 0.2
	Humifirst	51.4 ± 1.4	4.8 ± 0.2	21.4 ± 2.6	918 ± 36	2.0 ± 0.4
	Acadian	49.6 ± 1.3	5.6 ± 0.2	20.7 ± 1.8	882 ± 13	2.3 ± 0.2
	SilicaPower	53.9 ± 1.8	5.4 ± 0.1	23.8 ± 2.1	969 ± 39	2.5 ± 0.6
	CropSet	55.3 ± 1.8	5.8 ± 0.3	22.35 ± 2.6	957 ± 51	2.8 ± 0.2
	Mean	52.3	5.4	20.81 A	932	2.4
Drought	Control	51.7 ± 1.3	4.9 ± 0.1	13.3 ± 4.2	938 ± 33	2.5 ± 0.2
	Vesta	52.1 ± 1.3	5.2 ± 0.4	15.4 ± 2.3	966 ± 20	2.0 ± 0.0
	Humifirst	54.8 ± 2.2	5.5 ± 0.2	18.4 ± 2.1	951 ± 62	2.3 ± 0.2
	Acadian	51.2 ± 1.0	5.3 ± 0.2	18.1 ± 2.9	904 ± 19	2.8 ± 0.4
	SilicaPower	54.1 ± 3.0	5.5 ± 0.4	18.2 ± 4.2	939 ± 31	2.8 ± 0.4
	CropSet	46.1 ± 2.3	4.0 ± 0.4	19.9 ± 3.3	842 ± 33	1.8 ± 0.2
	Mean	51.6	5.0	17.22 B	923	2.3
P-value	D	0.6450	0.0947	0.0005	0.6794	0.5299
	BS	0.3899	0.5271	0.0087	0.4497	0.7134
	D x BS	0.0539	0.0384	0.502	0.4308	0.2607

Data are means of four replicates per treatment ± standard deviation. The plants were stressed for 7 days, then watered for 4 days, and the plant mass harvested. Data represent the means ± standard deviation (n = 4). A two-way ANOVA was used to evaluate the main effects of drought and biostimulant treatment, as well as their interaction. Different capital letters indicate significant differences between non-drought and drought stress conditions averaged across biostimulant treatments based on post hoc comparisons (LSD test) at P = 0.05.

Total dry biomass was significantly reduced under drought stress (*p* = 0.0005), and BS application also affected total plant dry biomass (*p* = 0.008). No significant drought × BS interaction was observed for total dry biomass (*p* = 0.5) (**Table 2**).

### 3.7 Tuber development and quality

**Figure 6:**
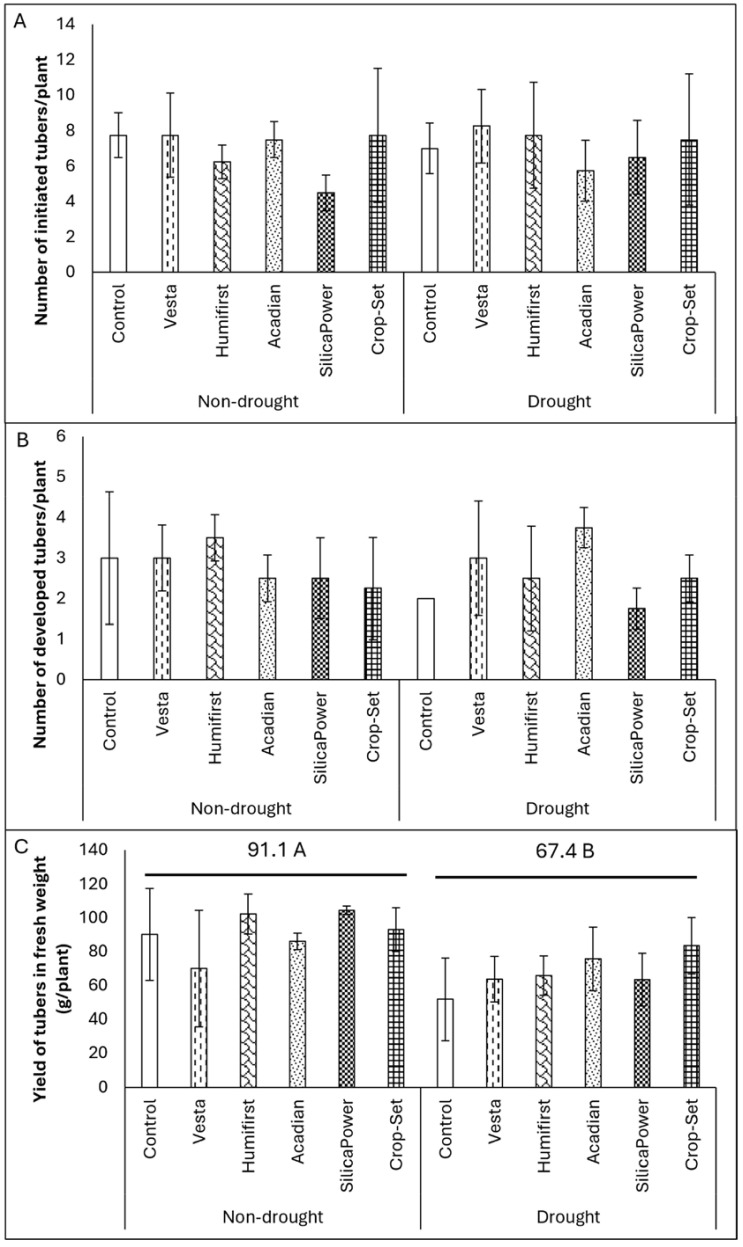
The effect of biostimulants on tuber development **(A, B)** and tuber yield **(C)** of potato plants after 7 days of drought stress, relative to non-drought-treated plants. The plants were treated with Vesta, Humifirst, Acadian, SilicaPower, and Crop-Set before the onset of drought stress. Following the drought stress, the plants were watered for 4 days, and then the tubers were harvested. Data represent the means ± standard deviation (n = 4). A two-way ANOVA was used to evaluate the main effects of drought and biostimulant treatment, as well as their interaction. Horizontal bars with different capital letters indicate significant differences between non-drought and drought stress conditions averaged across biostimulant treatments based on post hoc comparisons (LSD test) at P = 0.05.

The number of initiated and developed tubers did not differ after the recovery period (*p* = 0.7 and *p* = 0.4). Similarly, the application of BS did not result in significant changes in these parameters (*p* = 0.3 and *p* = 0.1) (**Figures 6A, B**). Moreover, the interactions between drought stress and BS were non-significant for these parameters (**Table 3**). Tuber yield decreased by 26% after recovery (*p* = 0.0002). However, BS did not affect tuber yield (*p* = 0.2), and the interaction between drought stress and BS was non-significant (*p* = 0.2) (**Figure 6C**).

**Table 3:** The effect of biostimulants on tuber dry matter, sugars and starch concentration of non-drought-stressed and drought-stressed plants.

Drought stress	Biostimulants	Dry matter		Glucose		Fructose		Sucrose	Total sugars		Starch	
D	BS	g 100 g^-1^		g 100 g^-1^		g 100 g^-1^		g 100 g^-1^				
Non-drought	Control	16.73 ± 1.5	abc	1.22 ± 0.20	abc	0.32 ± 0.08	ab	0.84 ± 0.25	2.40 ± 0.30	bcde	15.0 ± 1.9	bc
	Vesta	15.37 ± 0.8	c	1.50 ± 0.19	abc	0.46 ± 0.32	a	0.61 ± 0.28	2.59 ± 0.30	ab	12.1 ± 2.6	c
	Humifirst	16.19 ± 1.7	bc	1.11 ± 0.20	bcd	0.25 ± 0.05	b	0.75 ± 0.20	2.12 ± 0.26	cde	13.5 ± 3.2	bc
	Acadian	17.49 ± 1.8	abc	1.12 ± 0.20	bcd	0.30 ± 0.05	ab	0.78 ± 0.23	2.21 ± 0.23	bcde	14.3 ± 2.5	bc
	SilicaPower	17.68 ± 1.8	abc	1.13 ± 0.24	bcd	0.30 ± 0.04	ab	0.62 ± 0.18	2.06 ± 0.16	de	16.5 ± 1.7	ab
	Crop-Set	17.67 ± 1.8	abc	1.21 ± 0.21	abc	0.35 ± 0.13	ab	0.78 ± 0.35	2.35 ± 0.44	bcde	16.8 ± 3.8	ab
	Mean	16.9		1.21 A		0.33		0.73 B	2.29		14.7	
Drought	Control	16.3 ± 1.1	bc	1.45 ± 0.47	abc	0.48 ± 0.32	a	1.09 ± 0.34	3.04 ± 0.55	ab	14.4 ± 3.7	bc
	Vesta	16.3 ± 1.2	bc	1.06 ± 0.21	cde	0.31 ± 0.20	ab	1.12 ± 0.21	2.50 ± 0.26	bcd	13.9 ± 1.1	bc
	Humifirst	18.6 ± 2.1	ab	0.73 ± 0.13	e	0.17 ± 0.07	b	1.18 ± 0.18	2.08 ± 0.28	cde	15.0 ± 1.1	bc
	Acadian	17.1 ± 0.1	abc	0.84 ± 0.12	de	0.21 ± 0.07	b	1.17 ± 0.08	2.24 ± 0.19	bcde	14.7 ± 1.0	bc
	SilicaPower	19.2 ± 3.2	a	0.96 ± 0.19	cde	0.19 ± 0.10	b	1.38 ± 0.07	2.53 ± 0.29	bc	16.3 ± 5.1	ab
	Crop-Set	19.0 ± 1.3	a	0.72 ± 0.22	e	0.19 ± 0.02	b	1.03 ± 0.10	1.95 ± 0.28	e	19.4 ± 2.3	a
	Mean	17.8		0.96 B		0.26		1.16 A	2.39		15.6	
P-value	D	0.0844		0.0011		0.0670		0.0000	0.2752		0.2738	
	BS	0.0422		0.0050		0.0348		0.8646	0.0034		0.0178	
	D x BS	0.5539		0.0677		0.2089		0.2768	0.0330		0.8588	

Data are means of four replicates per treatment ± standard deviation. The plants were stressed for 7 days, then watered for 4 days, and then the tubers were harvested. Tubers of a comparable size within BS were analyzed (see **Supplementary Table 2**). Data represent the means ± standard deviation (n = 4). A two-way ANOVA was used to evaluate the main effects of drought and biostimulant treatment, as well as their interaction. Different capital letters indicate significant differences between non-drought and drought stress conditions averaged across biostimulant treatments based on post hoc comparisons (LSD test) at P = 0.05. Different lowercase letters indicate significant differences among treatment combinations (drought × biostimulants) based on post hoc comparisons (LSD test) at P = 0.05.

Dry matter, fructose, total sugars and starch remained unaffected by drought stress after recovery (**Table 3**). However, glucose concentration was decreased by 21% after recovery period (*p* = 0.001). In contrast, the sucrose level was 59% higher after these same conditions (*p* = 0.0000). When compared with the control, none of the applied BS produced significant variations in the levels of dry matter, glucose, fructose, sucrose, total sugars, and starch under non-drought conditions (**Table 3**). In contrast, under drought stress conditions, the BS showed different trends, with SilicaPower (18% increase) and Crop-Set (16% increase) significantly increasing the dry matter concentration in potato plants as compared to control (without BS). The glucose levels declined by 27, 50, 42, 34%, and 50% following Vesta, Humifirst, Acadian, SilicaPower, and Crop-Set treatments, respectively, as compared to control (without BS). This decline was 65, 56, 60, and 60% in fructose content when the plants were treated with Humifirst, Acadian, SilicaPower, and Crop-Set, respectively, as compared to control (without BS). Although the total sugars were not influenced by the drought stress alone, a significant interaction between drought and BS (p = 0.03) was observed, and total sugars were reduced by 32, 26, and 36% in the cases of Humifirst, Acadian, and Crop-Set, respectively, as compared to control (without BS). Conversely, the only change in starch concentration was an increase by 34% when potato plants were exposed to Crop-Set application, as compared to control (without BS) (**Table 3**).

## 4 Discussion

### 4.1 Water use efficiency

Drought stress is a common adverse factor limiting plant growth. It has a significant negative effect on the growth and development, morphology, and physiology of plants (Bashir et al., 2021; Ghadirnezhad Shiade et al., 2023). Similar results were observed in this study, where the phenology, physiology, and yield of potato were negatively impacted by drought stress (**Figure 1**). At the same time, it has been extensively reported that the adverse impacts of drought stress can be minimized by using plant BS, which can increase tolerance to drought stress in various plant species (Anjum et al., 2011; Alenazi et al., 2016; Nyawade et al., 2020). However, no substantial improvement was induced by the application of BS in the morphology and phenology of the potato plants (**Figure 1**). Unlike our findings, earlier studies have reported that the morphological appearance of the potato was improved after BS application (Cortiello et al., 2024).

In this experiment, the existence of drought stress was evidenced by the decline in leaf relative water content and increase in leaf temperature of the potato plants following drought stress (**Figure 2**). As a fundamental drought stress indicator, the leaf relative water content plays a key role in understanding plant growth and development since it closely reflects tissue water balance, which directly affects stomatal function, photosynthesis, and cell expansion (Yan et al., 2016). It was reported that leaf relative water content considerably declined following water-deficit conditions in spinach, and the application of seaweed extract (*Ascophyllum nodosum*) as a BS improved it (Xu and Leskovar, 2015). In this study, the potato plants showed inhibited leaf relative water content when they were exposed to drought stress; however, it was not affected by the application of BS under normal and drought stress conditions. This unchanged leaf relative water content suggests that osmotic adjustment was not sufficiently enhanced by the applied BS under the conditions of this study. In addition, plant water status was likely influenced by stomatal regulation and associated gas-exchange dynamics, rather than osmotic adjustment alone. Conversely, the intrinsic and instantaneous WUE increased following BS application (**Figure 2**), reflecting changes in the relationship between carbon assimilation and water loss. However, this increase was primarily driven by reductions in stomatal conductance and transpiration, accompanied by a decrease in photosynthetic rate, rather than by improvements in photosynthetic performance. Under such conditions, the increase in WUE largely represents a ratio-driven (apparent) response rather than a functional improvement in physiological efficiency or carbon gain. Such responses are characteristic of stress-induced stomatal limitation, where reduced water loss leads to higher WUE values without enhancing carbon assimilation (Medrano, 2002; Flexas et al., 2004). Therefore, the observed increase in WUE should be interpreted as a conservative water-saving strategy rather than an active enhancement of plant performance. This distinction is important, as higher WUE under stress does not necessarily translate into improved productivity (Blum, 2009). This is supported by the absence of corresponding increases in photosynthesis, biomass, or yield in the present study. Our results are therefore partially consistent with the first hypothesis, in that BS influenced WUE and gas exchange, however, this effect reflects apparent (ratio-driven) increases in WUE rather than functional improvements in carbon assimilation per unit water lost. This was particularly evident for Vesta, Humifirst, and SilicaPower under drought-stressed conditions and Acadian under non-drought conditions. While previous studies have reported improvements in WUE following BS application conditions (Alenazi et al., 2016; Nyawade et al., 2020; Kołodziejczyk, 2021), our findings suggest that such responses may depend on whether WUE increases are associated with enhanced photosynthetic capacity or primarily driven by stomatal limitation under stress.

### 4.2 Photosynthetic efficiency and performance

In the current study, drought stress induced a considerable reduction (29%) in the net photosynthetic rate of potato plants (**Figure 3**), and this inhibition was also reflected in the tuber yield (**Figure 6C**). Similarly, it was also reported that water-deficit conditions decreased the photosynthetic efficiency of potato plants by altering the balance between light energy absorption and photosynthetic electron transport within chloroplasts (Jacques et al., 2020; Gervais et al., 2021). The reason for inhibited photosynthetic efficiency under drought stress may be due to the overproduction of ROS in chloroplasts, which are involved in the degradation of chlorophyll molecules, thereby reducing the photosynthetic rate (Anjum et al., 2011, Sah and Sofo, 2024). Furthermore, drought stress can limit CO_2_ uptake into the leaves, reducing CO_2_ availability at carboxylation center, and consequently reduces photosynthetic efficiency (Li et al., 2017). BS have been reported to mitigate drought-induced limitations of photosynthesis by enhancing antioxidant defenses, modulating stomatal behavior, and promoting the accumulation of osmolytes such as proline (Franzoni et al., 2022). However, we found contrasting results to the literature, as the application of various commercial BS could not enhance the photosynthetic efficiency of potato plants (**Figure 3**). This apparent discrepancy may be explained by strong stomatal limitation under drought, as indicated by reduced stomatal conductance and intercellular CO_2_ concentration. Under such conditions, carbon assimilation is mainly constrained by limited CO_2_ diffusion rather than biochemical processes. Consequently, any protective effects of BS may not translate into increased photosynthetic rates. The lack of changes in chlorophyll fluorescence further suggests that photochemical efficiency was not improved. Together, these results indicate that BS primarily influenced water use strategies rather than directly improving photosynthetic capacity, consistent with the observed increase in WUE without corresponding gains in photosynthesis. Moreover, the variability in BS efficacy may be related to the diverse nature of these products, which differ in composition, mode of action, and application strategy, thereby resulting in variable physiological responses across studies.

One of the initial and most common physiological responses of plants to water stress is a reduction in stomatal conductance (Medrano, 2002). We also found a considerable decrease in stomatal conductance (70%) upon exposure to water-deficit conditions (**Figure 3**). Water scarcity leads to reduced photosynthetic efficiency, primarily due to stomatal closure that limits the availability of CO_2_ for carbon fixation in the Calvin cycle (Gupta et al., 2020). Stomatal closure results in a decrease in both transpiration rate and photosynthesis. Drought leads to a reduction in the carbon assimilation rate due to the limitation of stomatal CO_2_ diffusion. It has been documented that drought-induced stomatal limitations altered the metabolic pathways in plants through reduced CO_2_ and nutrient uptake (Laura Fernández-de-Uña 2025; Pinheiro and Chaves, 2011). However, the application of BS resulted in improved stomatal conductance under drought stress (Liatile et al., 2022; Mansour et al., 2023). Similarly, it was reported that following the application of a liquid organic amendment containing amino acids and organic matter, an improvement in stomatal conductance in spinach was observed under drought stress (Ekinci et al., 2015). However, in the current experimental system, the application of BS could not induce an increase in stomatal conductance and other photosynthesis-related metrics (with rare exceptions) (**Figure 3**). This limited physiological response may partly explain the absence of significant changes in total yield upon exposure to BS, this may reflect the short duration of the experiment and the early developmental stage at which drought stress was imposed, rather than a definitive absence of BS effects on yield formation. Contrarily, earlier studies found that supplementing different BS resulted in enhanced performance of gas exchange parameters in various plant species, leading to improved plant growth under both normal and stress conditions (Arafa et al., 2011; Gururani et al., 2013).

Chlorophyll fluorescence is a widely used indicator for assessing the impact of drought stress on photosynthesis, as it reflects changes in photosynthetic efficiency, particularly in electron transport rate and photosystem II yield (Genty et al., 1989; Chen et al., 2023). Generally, both parameters exhibit a decline in response to drought stress. A similar pattern was observed in the electron transport rate and photosystem yield in this experiment (**Figure 4**). The reason behind their decline might be the reduced CO_2_ availability and protective downregulation of photosynthesis to prevent photodamage (Zivcak et al., 2013), although other factors such as metabolic limitations and stress-induced damage to the photosynthetic apparatus may also contribute. Like other parameters related to photosynthesis, the application of BS could not improve the electron transport rate and photosystem yield in potato leaves. This discrepancy may be explained by the strong stomatal limitation under drought conditions, which restricted CO_2_ availability and likely constrained downstream photochemical processes.

Leaf pigments, notably chlorophyll index and flavonol index, are sensitive plant indicators in response to drought stress (Daryanavard et al., 2023; Vijayalakshmi et al., 2024). In the present study, the chlorophyll index remained unaffected by drought stress, whereas flavonol index decreased under drought conditions, accompanied by an increase in the nitrogen balance index (**Figure 5**). Similar responses have been reported previously, where chlorophyll index was not altered by drought stress, while flavonol content declined by 36% compared to control plants (Mircea et al., 2023). In contrast, water-deficit conditions have been shown to induce a progressive increase in nitrogen balance index, consistent with the patterns observed in our study. The decrease in flavonol index under drought conditions may be explained by multiple factors. For instance, drought-induced oxidative stress may lead to degradation of flavonol compounds, while reduced carbon assimilation under stomatal limitation could constrain the biosynthesis of secondary metabolites. In addition, developmental stage effects during early tuber initiation and bulking may influence flavonol accumulation patterns. Methodological aspects related to optical index measurements may also contribute to the observed responses. The reduction in flavonol index is likely due to a higher utilization of flavonol under stress, which is the reason for the higher nitrogen balance index. It is extensively reported that the application of BS (extracts from *Artemisia vulgaris* L.) led to an increase in chlorophyll (a and b), carotenoids, and polyphenols concentration (Findura et al., 2020). Contrary to the literature, no improvement in leaf pigments was observed by the supplied BS (**Figure 5**). This may be attributed to a stomatal limitation under drought conditions, which likely constrained the synthesis and accumulation of secondary metabolites such as flavonols. In addition, the unchanged chlorophyll index and lack of improvement in photosynthetic performance suggest that the tested BS did not substantially influence the photochemical systems under the conditions of this experiment. These responses may also reflect the short duration of stress exposure and the absence of measurable changes in plant nutrient status, which can play a key role in pigment regulation.

### 4.3 Plant morphology, tuber development and quality

The growth variables (measured after recovery period) of potato remained unaffected drought stress, indicating that the plants were able to recover effectively from stress conditions (**Table 2**). Similarly, in another report, the potatoes recovered well after the recovery period (Li et al., 2023). Regarding the BS, our results showed no effect on growth of potato which is in contradiction to the previous study on potato in which the BS (based on plant flavonoids) led to improved tuber size, tuber yield and marketable yield of three potato varieties (Maris Piper, Navan, Lady Rosetta) (Salvage et al., 2024). The growth parameters of potato were also improved following the application of extracts from brown seaweed *Ascophyllum nodosum*, aloe vera leaf, garlic bulb, and moringa leaf (Mbuyisa et al., 2023). It was also found that the application of humic acid as a BS could enhance the vegetative growth (Alenazi et al., 2016). This discrepancy may be related to differences in BS dose, formulation, and application strategy.

Yield is a crucial indicator of productivity in potato cultivation, and it is significantly threatened by drought stress (Nasir and Toth, 2022). Similarly, drought stress resulted in a 26% reduction in tuber yield (determined after recovery period), which may be attributed to the cumulative effect of early stress on resource allocation. While BS did not induce significant change in yield or related parameters (**Figure 6**), contrasting effects have been reported previously (Barbaś et al., 2025). For instance, the application of humic acid as a BS could enhance the vegetative growth, tuber weight, yield, and tuber quality at harvest (Alenazi et al., 2016). In addition, a BS named Supporter (made of amino acids) improved yield and quality of four potato varieties (Innovator, Lilly, Lady Claire, and Verdi) (Barbaś et al., 2025). However, such responses may depend on environmental conditions, particularly soil characteristics, which have been shown to influence BS performance) (Barbaś et al., 2025). Therefore, the absence of growth improvement in the present study may reflect context-specific factors, rather than a lack of efficacy of the tested BS.

The drought stress did not induce any considerable variations in the concentrations of dry matter, fructose and total sugars of potato tubers (assessed after recovery period) (**Table 3**). It may be due to the physiological plasticity of plants, as shown during the recovery period, which allows them to recover their metabolic functions (Xu et al., 2010; Lemoine et al., 2013). Although the decline in glucose levels was observed, sucrose levels improved after the recovery period, indicating that the upregulated sucrose levels may aid in restoring cellular functions and growth.

After the recovery period, SilicaPower and Crop-Set were able to enhance dry matter, while Crop-Set also improved starch content (**Table 3**). These responses indicate the formation of denser, more carbon-rich tuber tissue, however, such changes may reflect drought-induced concentration effects rather than active changes in carbon allocation. In this context, BS application appeared to influence tuber composition without a corresponding increase in early biomass accumulation or yield. This suggests that BS may influence tuber composition (e.g., dry matter, starch, and soluble sugars), although no direct evidence of altered carbon allocation is provided. Similar to these findings, humic substances (Agriful) and beneficial bacteria (Groundfix^®^) applied by drip irrigation were found to enhance tuber yield, starch, dry matter, and vitamin C in potato (Andrejiová et al., 2023). In our study, sugar concentrations (determined after recovery period) were inhibited when potato plants were exposed to BS under drought stress conditions (**Table 3**). This response may reflect changes in carbohydrate utilization or concentration effects rather than direct changes in carbon allocation. Unlike our findings, a previous study reported a significant increase in total sugars, reducing sugars, and sucrose contents of potatoes after the application of Asahi SL^®^ (para-nitrophenolan sodium, ortho-nitrophenolan sodium, 5-sodium nitroguaiacolan) BS (Zarzecka and Gugała, 2018). In another study on tomatoes, a significant improvement was observed in sugar content after the application of legume-derived protein hydrolysate Trainer (Rouphael et al., 2017).

Taken together, it is demonstrated that while drought stress had a limited effect on carbohydrate metabolism (assessed after recovery period), the application of BS led to differential metabolic responses. Specifically, the enhancement in dry matter and starch content with SilicaPower and Crop-Set indicates changes in tuber composition, rather than a demonstrated increase in drought tolerance in potato. These changes may reflect alterations in carbohydrate accumulation or concentration effects under drought conditions. However, the decline observed in sugar-related traits indicates a complex and parameter-specific response that warrants further investigation.

### 4.4 Responses of potato to drought and biostimulants

The demonstration of drought stress in potato plants was evident from their stunted phenological development and a reduction in leaf relative water content. This water limitation reduced photosynthetic efficiency, reflecting the direct physiological impact of drought stress. Under drought stress, yield and glucose decreased, whereas sucrose increased, while other morphological traits and tuber development and quality parameters remained largely unchanged after recovery period. Although some of the commercial BS (Vesta, SilicaPower, Humifirst) increased WUE under drought conditions, suggesting a more conservative gas−exchange strategy that improves the WUE instead of improving the absolute rate of photosynthesis. At tuber level, changes in tuber composition by two of the five tested BS (SilicaPower and Crop-Set) towards higher dry matter and starch content suggests compositional changes rather than direct evidence of altered sink activity or osmotic adjustment.

Taken together, our findings indicate that the above-mentioned BS influenced potato drought responses primarily through two physiological pathways rather than through direct stimulation of plant growth. First, most of the BS improved WUE by promoting a conservative gas-exchange strategy, reducing water loss relative to carbon assimilation under drought conditions. Second, some BS were associated with changes in tuber composition, including increased dry matter and starch accumulation without a corresponding increase in total biomass. These findings indicate that the effects of BS in this experiment were mainly associated with resource-use efficiency and metabolic allocation strategies, rather than broad range of drought stress tolerance. The observed combination of conservative gas exchange (increased WUE) and changes in tuber composition is consistent with efficiency mechanisms described for drought tolerance in potato, where improved WUE and carbon partitioning have been highlighted as important components of adaptation to water deficit (Obidiegwu, 2015; Aliche et al., 2020).

Apart from the two processes discussed above, through which some of the BS may contribute to specific aspects of drought tolerance, this study did not reproduce the growth−enhancing and stress−ameliorating effects reported by other studies. This suggests that BS responses are context dependent and influenced by factors such as soil type, climate, formulation, and application strategy. In addition, differences in BS composition and application methods (e.g., foliar versus soil application) may induce distinct physiological responses and should be considered when comparing treatments. However, the comparison among BS was not intended to evaluate the effect of different application methods. Instead, each BS was assessed according to its recommended use, including the prescribed rate and method of application. In the current experiment, we applied the BS at concentrations, application rates and frequencies according to commercial guidelines and tested them under greenhouse conditions designed to mimic field production, thereby testing in a realistic setting close to farming conditions. However, despite this practical relevance, the interpretation of the results should be considered within the specific experimental context. In particular, the short duration of drought (7 days) and recovery (4 days), together with the early growth stage assessed, primarily capture short-term physiological responses rather than long-term effects on biomass and tuber development. Furthermore, as root traits, nutrient dynamics, and biochemical or hormonal processes were also not evaluated, therefore the observed changes in WUE and tuber composition cannot be attributed to specific mechanisms and should be interpreted with caution.

## 5 Conclusion

This study provides a comprehensive evaluation of commercial BS during early growth stages of potato, with a focus on leaf-level physiology, WUE, and tuber quality and yield at tuber initiation under drought and non-drought conditions. Drought stress substantially impaired leaf water status, photosynthetic performance, and tuber yield, confirming the sensitivity of potato to short-term water limitation at the phenological stage. Most morphological and tuber quality traits recovered after post-drought rewatering, with the exception of changes in individual carbohydrate components. The tested commercial BS did not improve growth parameters including plant dry biomass or tuber yield under either optimal or drought conditions, but they induced selective physiological and compositional responses, particularly under drought stress. For instance, Vesta and SilicaPower increased intrinsic WUE by 36% and 30%, respectively, while Vesta, Humifirst, and SilicaPower increased instantaneous WUE by 25%, 25%, and 33%, respectively. In addition, SilicaPower and Crop−Set enhanced tuber dry matter concentration by 18% and 16%, and Crop−Set further increased starch concentration by 34% under drought. These responses suggest that BS effects under short-term drought are primarily associated with two processes: processes: (i) improved WUE through modified gas-exchange behavior, and (ii) changes in tuber composition consistent with altered carbon allocation to storage compounds. However, given that the experiment was limited to early growth stages up to tuber initiation, these findings should be interpreted as short-term physiological responses rather than indicators of whole-season yield performance or drought resilience. Future research should validate these findings under longer growth durations and field conditions and further elucidate the underlying mechanisms by which specific BS modulate WUE and tuber carbon allocation under drought.

## Data Availability

The original contributions presented in the study are included in the article/**Supplementary Material**. Further inquiries can be directed to the corresponding author.
